# Investigation of Serum Pregnancy-Specific Beta-1-Glycoprotein and Relationship with Fetal Growth Restriction

**DOI:** 10.5935/1518-0557.20210068

**Published:** 2022

**Authors:** Sabiha Tuzluoğlu, Emin Üstünyurt, Süleyman Serkan Karaşin, Zeynep Toksoy Karaşin

**Affiliations:** 1 Obstetrics and Gynecology, Health Sciences University Bursa Yüksek İhtisas Training and Research Hospital, Bursa, Turkey

**Keywords:** fetal growth restriction, serum beta 1 glycoprotein, pregnancy

## Abstract

**Objective:**

The most used definition for fetal growth restriction (FGR) is a fetus whose estimated weight is below the 10^th^ percentile for its gestational age. Pregnancy-specific beta-1-glycoprotein (PSG-1) is an immunomodulator found in maternal serum during pregnancy. This study aimed to determine the serum levels of PSG-1 and clarify the potential role of this molecule in the etiopathogenesis of FGR.

**Methods:**

Eighty women carrying fetuses with FGR and 80 healthy pregnant women were included in the study. Demographic data, laboratory values, and Doppler Ultrasonography (USG) results of all cases were recorded. Venous blood samples were taken from all cases before birth. PSG-1 values were studied by the ELISA method. An Independent Samples T-test was used to evaluate the results. The correlations between parameters were evaluated based on Spearman's rank correlation coefficient. *P*-values <0.05 were considered statistically significant.

**Results:**

When the groups were evaluated for serum PSG-1 levels, the median serum PSG-1 level was lower in pregnant women carrying fetuses with FGR than in controls (0.05 < *p*>0.10). Median serum PSG-1 was lower in patients with absent end diastolic flow (AEDF) in the umbilical artery in Doppler ultrasound scans than in patients without AEDF, but the difference was not statistically significant (*p*>0.05). In patients with serum PSG-1 values below 12.93 with 50% sensitivity and 76% specificity, the risk of FGR was higher.

**Conclusions:**

Serum PSG-1 levels may be lower in complicated pregnancies due to problems related to placental insufficiency and FGR.

## INTRODUCTION

Fetal growth restriction (FGR) is defined as a fetus with an estimated weight below the 10th percentile for its gestational age (Dubinsky & Sonneborn, 2020; Clausson *et al*., 2001). Although FGR is a complication seen in 5-10% of all pregnancies, the perinatal period after a preterm birth is a time of increased mortality and morbidity in childhood (Say *et al*., 2003; Resnik, 2002).

Neonatal outcomes of FGR include perinatal asphyxia and neonatal adaptive problems, and short- and long-term sequelae. In infants with FGR, the perinatal mortality rate is 10-20 times greater than the rate observed in healthy fetuses (Barker, 1999). Perinatal mortality and morbidity can decrease by correct diagnosis, appropriate surveillance, and timely intervention. Since successful intrauterine treatment has not been defined for fetuses with FGR, planning delivery for the most appropriate time is essential in antenatal management. In timing birth, the aim is to ensure that maximum gestational age is reached and the risks to intrauterine life are minimized (Clausson *et al*., 2001; Soothill *et al*., 1987). Pregnancies with fetal growth retardation are of high risk and may become a public health problem due to poor fetal and neonatal outcomes. It is essential to identify and adequately follow fetuses with FGR, since this complication negatively affects perinatal outcomes in terms of mortality and morbidity. For this reason, many studies have been conducted for diagnostic purposes. However, to date, no useful biochemical marker or endocrine test has been found to monitor fetal growth or predict growth retardation healthily.

Maintenance of intrauterine development is possible with adequate oxygen and nutritional support. This condition happens with the continual change and development of uteroplacental circulation throughout pregnancy (Macara *et al*., 1996)though an alternative explanation is that the placenta fails to adequately transfer oxygen to the fetus from the intervillous space. Because oxygen transport takes place within the terminal villi, we undertook the first detailed studies of villous ultrastructure structure and immunohistochemistry in order to determine the likely origin of fetal hypoxia in this condition. Terminal villi were examined ultrastructurally using transmission electron microscopy and by immunohistochemical localization of matrix molecules (laminin and collagens I, III and IV. The placenta provides nutritional and respiratory support for the maintenance of fetal life. Therefore, disorders in placental structure and function are responsible for FGR (Salafia *et al*., 1995). Molecules that effectively ensure uteroplacental circulation are thought to contribute to fetal development. Many published studies have looked into this concept. However, none found diagnostic markers of FGR or described its pathophysiology clearly.

Pregnancy-specific beta-1-glycoprotein (PSG-1) is a protein subgroup recently isolated from human placenta and maternal serum (Grudzinskas *et al*., 1983; Tatarinov & Masiukevich, 1970) Bohn found that PSG was immunologically similar to Schwangerschafts protein 1 (SP1), which the author had isolated from human placenta (Bohn, 1971; Chou & Plouzek, 1992). Serum PSG-1 levels were used to diagnose pregnancy and pregnancy-related complications (Chou & Plouzek, 1992; Tamsen *et al*., 1983). During pregnancy, PSG-1 becomes detectable in maternal serum 10 to 18 days after the ovulatory luteinizing hormone surge. It increases as pregnancy progresses and doubles within 2.4 days and achieves a very high level of 200-400µg/ml at term (Lee *et al*., 1979; Lenton *et al*., 1981). Besides, PSG-1 is used as a marker for malignant disease and in the monitoring of choriocarcinoma therapy (Tatarinov & Masiukevich, 1970). Molecular studies also revealed the substantial similarity of human PSG species with carcinoembryonic antigen (CEA) members. Carcinoembryonic antigen is a widely used marker for colonic cancers (Shively & Beatty, 1985).

Pregnant women were through to secrete serum pregnancy-specific beta-1-glycoprotein (PSG-1). It is the most released fetal protein from placental syncytiotrophoblasts to the maternal circulation during late pregnancy (Zhou *et al*., 1997). Studies focused mostly on the effect of this molecule on the regulation of the maternal immune system.

This study aims to compare the serum concentrations of PSG-1 seen in healthy pregnant women and in pregnant women carrying fetuses with growth restriction, and to identify patients at risk of FGR by assessing the relationship between this molecule and pregnancies complicated by intrauterine growth retardation. The goal is to contribute to the adoption of strict control and follow-up measures to ultimately decrease fetal and maternal mortality and morbidity caused by FGR.

## MATERIAL AND METHODS

This cross-sectional study was conducted in the Gynecology and Obstetrics Department of the Bursa Yüksek İhtisas Training and Research Hospital between March 2018 and December 2018. It included 160 volunteers, of which 80 were patients diagnosed with FGR and 80 were healthy individuals assigned to the control group. The local Ethics Committee approved the study and assigned it certificate number 2011-KAEK-25 2018 / 03-05.

### Determination of Groups

According to the recommendations of the Royal College of Obstetricians and Gynecologists (RCOG) from 2013 for the diagnosis of FGR, pregnant women carrying fetuses with an estimated fetal weight (EFW) <10 p, oligohydramnios, abnormal umbilical artery in Doppler ultrasound (absent end diastolic flow or reverse flow) or an EFW <3p are diagnosed as having fetuses with FGR.

Obstetric examinations were performed, and the length of pregnancy in weeks was established based on the last menstrual period and confirmed by the first trimester ultrasound examination. The number of weeks of gestation considered in the study was calculated based on the last menstrual period and confirmed based on early ultrasound measurements. Birth weight of the newborns was recorded after delivery.

The EFW calculated by ultrasound of the fetuses carried by healthy women was above the 10^th^ percentile (n=80). Subjects with an EFW below the 10^th^ percentile were included in the study group and diagnosed with intrauterine growth restriction (n=80). A single operator performed color Doppler measurements using a Voluson 730 Pro Color Doppler ultrasonography device. A 3.5 MHz convex probe was used in examinations. We evaluated the systole diastole ratio (S/D) and end diastolic flow in the umbilical artery in Color Doppler.

None of the pregnant women included in our study had a poor obstetric history, maternal or fetal problem. History of multiple pregnancy; chronic disease; infection causing FGR; smoking during pregnancy; use of cannabis or cocaine; and alcohol drinking were not studied.

### Collection of Blood Samples

Blood samples of approximately 5 ml were drawn from study participants at any time of the day to investigate PSG-1 levels. The samples taken from each volunteer participant were stored in biochemistry tubes containing an extra separator gel. After waiting for approximately 20 minutes at room temperature, the samples were centrifuged at 5000 rpm for 5 minutes and kept at -20 degrees. PSG-1 levels were studied with ELISA Awerenes Technology INC, Human Pregnancy Specific Beta 1 Glycoprotein assay kits. Results were reported in ng/ml. Complete blood count, blood glucose, blood urea nitrogen, creatinine, aminotransferase, and PSG-1 levels were evaluated.

### Statistical Evaluation

Statistical analysis was performed with Statistical Package for the Social Sciences (SPSS) version 22.0. The Kolmogorov-Smirnov test was used to test data distribution normality. We presented descriptive analyses using mean (±SD) for normally distributed variables and median (range) for data not following a normal distribution. In data comparisons, Student's t-test was used for normally distributed data and the Mann Whitney U test was used for data not following a normal distribution. Spearman's rank correlation was used to assess the relationships between quantitative data. Univariate logistic regression analysis was applied to determine the variables that might predict FGR. Variables with *p*<0.25 as a result of univariate analysis were included in multivariate analysis. The Backward LR method was used in multivariate analysis. The compatibility of the models with the data was evaluated with the Hosmer and Lemeshow test.

ROC curve analysis was used in the calculation of cutoff values for serum PSG-1 level. All statistical calculations were evaluated at a 95% confidence interval and significance was attributed to differences with *p*<0.05.

## RESULTS

In this study, 80 (50.0%) of 160 pregnant women were pregnant with fetuses with FGR and 80 (50.0%) were healthy controls. Frequencies and percentages related to pregnancy outcomes of the participants are described in Table 1.

**Table 1.** Pregnancy Results and Laboratory Data of All Pregnant Women Included in the Study (n=160).

Pregnancy Results and Laboratory Data	Mean±SD; Median (Range)
Age (y)	25.87±4.66
Body Mass Index (kg/cm^2^)	28.40±4.65
Gravidity (n)	2 (6)
Parity (n)	0 (4)
Abortion (n)	0 (4)
Gestational Age (w)	36.67±3.48
Birth Weight (kg)	2607.84±887.83
Serum PSG-1 (ng/ml)	16.51 (437.33)
Hemoglobin (g/dL)	11.73±1.28
Platelets (103/milliliters)	232 (330)
Blood Urea Nitrogen (mg/dL)	8.63±6.90
Creatinine (mg/dL)	0.64 (11.2)
Aspartate Aminotransferase (AST) (U/L)	22.08±8.43
Alanine Aminotransferase (ALT) (U/L)	13.68±7.56

SD: Standard Deviation. Descriptive analyses were presented using (Mean±SD) and Median (range) for normally distributed and non-normally distributed variables, respectively.

The mean age of the pregnant women included in the study group was 25.9±4.7, while the mean body mass index was 28.4±4.6. The mean length of gestation of the pregnant women included in the study was 36.6±3.4 weeks, and the mean birth weight of the babies was 2607.84 ± 887.83 grams (Table 1). Complete blood count and biochemical parameters of the pregnant women included in the study are given in Table 1.

The mean age of the pregnant women with FGR was higher than that of controls, but the difference was not statistically significant (*p*>0.05). The FGR group had a lower body mass index on average than the control group, but the difference was not statistically significant (*p*>0.05) (Table 2).

**Table 2.** Comparison of Study Groups by Demographic Features and Laboratory Data (n=160).

Demographic Features and Laboratory Data	FGR Group n=80	Control Group n=80	*p* value
	Mean±SD; Median (Range)	Mean±SD; Median (Range)	
Age *	26.21±4.83	25.53±4.50	0.353
Body Mass Index *	28.07±5.29	28.73±3.92	0.370
Gravidity β	2 (6)	2 (5)	0.947
Parity β	0 (3)	0 (4)	0.646
Abortion β	0 (4)	2 (2)	0.391
Gestational Week *	34.59±3.58	38.75±1.65	<0.001
Birth Weight (grams) *	1907.69±662.08	3308.00±394.03	<0.001
Hemoglobin* (g/dL)	11.90±1.18	11.57±1.36	0.097
Platelets β (103/milliliters)	237 (330)	227 (282)	0.569
BUN* (mg/dL)	9.60±9.43	7.67±2.25	0.077
Creatinine β (mg/dL)	0.61 (11.2)	0.66 (8.41)	0.011
AST* (U/L)	21.19±7.78	22.97±8.99	0.181
ALT* (U/L)	14.33±8.94	13.04±5.86	0.283

SD: Standard Deviation

* Student t test used for comparisons between groups

β Mann Whitney U test used in comparison between groups

We evaluated the groups in terms of gravidity and parity. We did not find a statistical difference between the groups (*p*>0.05). The FGR group had fewer weeks of gestation and newborns with lower birth weight than controls (*p*<0.05) (Table 2).

We did not find a statistically significant difference in hemoglobin, platelet, BUN, or aminotransferase levels between the groups (*p*>0.05). The creatinine levels of pregnant women with fetuses with FGR were statistically higher than the levels seen in controls (*p*<0.05) (Table 2).

The median PSG-1 level of pregnant women with intrauterine growth retardation was lower than that of controls. However, the difference was statistically limited (0.05 < *p*<0.10) (Table 3). We compared PSG-1 values in the FGR group based on umbilical artery Doppler ultrasound findings. In patients with absent end diastolic flow (AEDF) in the umbilical artery, median serum PSG-1 was lower when compared with women without AEDF, although the difference was not statistically significant (*p*>0.05) (Table 4).

**Table 3.** Comparison of Working Groups According to PSG-1 Values.

n=160	FGR Group n=80	Control Group n=80	*p* value
	Median (Range)	Median (Range)	
Serum Psg-1β (ng/ml)	13.48 (328.91)	18.33 (437.33)	0.058

SD: Standard Deviation

β Mann Whitney U test used in comparison between groups

**Table 4.** Comparison of FGR Groups according to Umbilical Artery Absent End Diastolic Flow in  Doppler Ultrasound in terms of PSG1 levels.

n=80	Absent End Diastolic Flow n=29	Without Absent End Diastolic Flow n=51	*p*-value
	Median (Range)	Median (Range)	
**Serum Psg-1β (ng/ml)**	12.56 (254.5)	14.05 (328.91)	0.964

SD: Standard Deviation

β Mann Whitney U test used in comparison between groups

Correlation analysis of the data from pregnant women with FGR found only a moderately significant correlation between age and FGR (*p*<0.05) (Table 5).

**Table 5.** Correlation of PSG1 and Other Parameters in FGR Groups (n=80).

Parameters	PSG-1 (ng/ml)
Age	r= -0.328
	*p*=0.003
BMI	r= -0.123
	*p*=0.277
Gestational Age	r= -0.039
	*p*=0.734
Birth Weight	r=0.009
	*p*=0.934
Hemoglobin	r= -0.013
	*p*=0.908
Platelets	r=0.162
	*p*= 0.152
Blood Urea Nitrogen	r= -0.016
	*p*=0.891
Creatinine	r= -0.083
	*p*=0.464
Aspartate Aminotransferase	r=0.029
	*p*=0.796
Alanine Aminotransferase	r= -0.027
	*p*=0.812

r: Spearman's rank correlation coefficient.

BMI: body mass index

Binominal logistic regression was used to determine the variables that might predict FGR. Univariate and multivariate logistic regression analyses were performed to determine the factors affecting FGR and their respective odds ratios. The control group was chosen as the reference category.

In the multivariate model created for pregnant women with FGR, birth weight was determined as a predictor of intrauterine growth restriction. As a result, when birth weight increases by one unit, the risk of intrauterine growth retardation decreases by 0.9% (Table 6).

**Table 6.** Logistic Regression Analysis for FGR Groups.

	Univariate Analysis Results				Multivariate Analysis Results			
	Wald	OR	CI	*p*	Wald	OR	CI	*p*
**Age**	0.871	1.032	0.965 - 1.104	0.351				
**BMI**	.811	0.969	0.906 - 1.037	0.368				
**Gestational Age**	36.35	0.443	0.340 - 0.578	**<0.001**				
**Birth Weight**	18.78	0.991	0.986 - 0.995	**<0.001**	18.87	0.991	0.986 - 0.995	**<0.001**
**Psg-1**	0.965	0.998	0.995 - 1.002	0.326				
**Hemoglobin**	2.729	1.233	0.962 - 1.582	**0.099**				
**Platelets**	0.152	1.000	1.000 - 1.000	0.697				
**Blood Urea Nitrogen**	4.789	1.149	1.015 - 1.301	**0.029**				
**Creatinine**	0.285	1.069	0.837 - 1.365	0.593				
**Aspartate Aminotransferase**	1.725	0.974	0.936 - 1.013	**0.189**				
**Alanine Aminotransferase**	1.100	1.025	0.979 - 1.072	0.294				

GA (95 %); BMI: body mass index; OR: odds ratio; CI: confidence interval; Wald: test statistic value.

Since the dependent variable consists of 2 groups, binominal logistic regression was used. Control group taken as reference category. As a result of univariate analysis, variables with *p*<0.25 were included in multivariate analysis. Backward LR was used in multivariate analysis. Hosmer and Lemeshow test *p*> 0.05 was out, and the models had good data compatibility.

According to logistic regression analysis results, serum PSG-1 did not affect the risk of intrauterine growth retardation in pregnant women (*p*>0.05) (Table 6).

**Figure 1:**
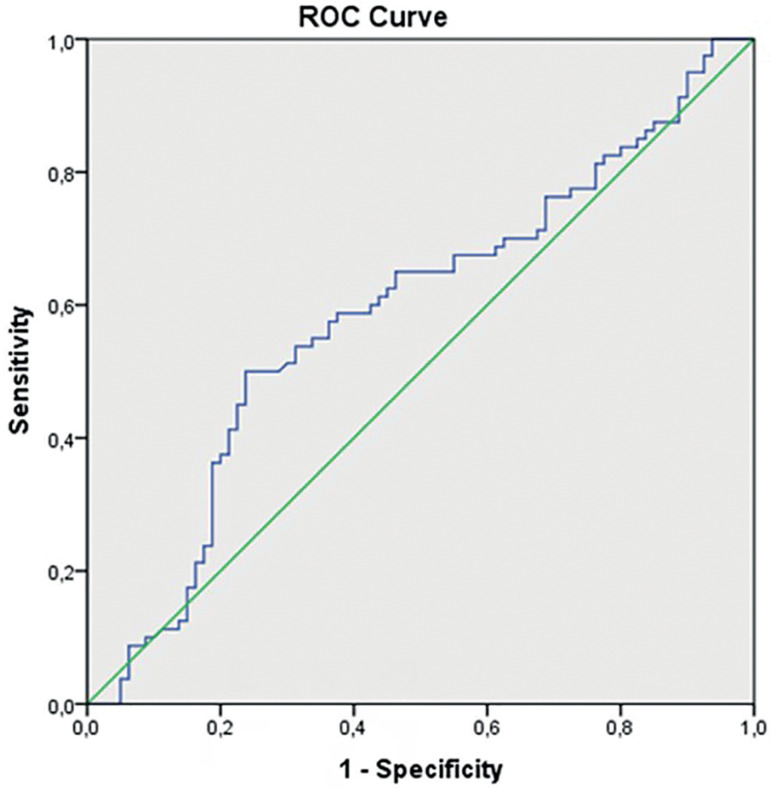
ROC Curve for Serum PSG-1 in Pregnant Women with Fetuses with Intrauterine Growth Restriction.

As a result of the 'Receiver Operating Characteristic' (ROC) analysis applied for serum PSG-1, the area under the process characteristic curve was calculated as 0.587 and was assigned limited significance (*p*=0.058). The cut-off value for serum PSG-1 was 12.93. In cases where serum PSG-1 value with 50% sensitivity and 76% specificity was below 12.93, the risk of intrauterine growth retardation was higher (Table 7) (Figure 1).

**Table 7.** Roc Analysis for Serum PSG-1 in FGR Group.

Area Under the ROC Curve (95% CI)	*p*	Cut-off	Sensitivity	Specificity
0.587 (0.498 - 0.676)	0.058	12.93	0.50	0.76

## DISCUSSION

Intrauterine growth restriction (retardation) occurs when the fetus lags behind the weight it has to reach for its calculated gestational age. The American College of Obstetricians and Gynecologists (ACOG) defines intrauterine growth retardation as having a fetus with an estimated fetal weight below the 10^th^ percentile for its gestational age (ACOG, 2013). We included pregnant women with fetuses with estimated fetal weights below the 10^th^ percentile in our study group.

Compared with fetuses with normal growth, fetuses with FGR are more predisposed to having asphyxia, meconium aspiration, respiratory distress syndrome, massive pulmonary hemorrhage, chronic lung disease, hypothermia, hypoglycemia, hypocalcemia, polycythemia, sepsis, intraventricular hemorrhage, necrotizing enterocolitis, and coagulation abnormalities (ACOG, 2013).

Numerous molecules circulating in maternal peripheral blood during pregnancy are potentially useful markers for pregnancies at risk of FGR. Information about the pathological mechanisms underlying some pregnancy-related diseases has increased significantly. However, most markers are not discriminatory enough to be used clinically and show relatively considerable overlap with levels in pregnant women with average obstetric outcomes (Reis *et al*., 2002; Page *et al*., 2002).

Various studies have found that changes in the placental bed have a role in the pathophysiology of FGR (Khong & Sawyer, 1991; Khong *et al*., 1986). In cases of intrauterine growth retardation, trophoblast invasion did not occur in the decidua or myometrial layers along some of the spiral arteries.

Pregnancy-specific beta-1-glycoprotein was detected in 1970 by Tatarinov and Masyukevich as a new protein in the serum of pregnant women (Tatarinov & Masiukevich, 1970; Chou & Plouzek, 1992). This molecule is a significant pregnancy-associated protein produced by the placenta. It is one of the oncogenic gene products usually examined as a potential marker for the early diagnosis of cancer and effectiveness of cancer therapy. Despite the uses of PSG in the diagnosis of pregnancy and malignancy, this protein's exact physiological role is unknown.

Studies have shown that PSG-1 belongs to a widely purified family of proteins secreted from placental syncytiotrophoblasts from the 2^nd^ week of pregnancy (Teglund *et al*., 1994). Human placental lactogen (HPL), PSG-1, and alpha-fetoprotein(AFP) values in imminent abortion prediction were investigated in a study performed by Hertz & Schultz-Larsen (1983). Low PSG-1 values were a better predictor of abortion than HPL and AFP. In another study, the serum PSG-1 levels of 82 healthy pregnant women were compared to the levels seen in 37 women with preeclampsia, eight with fetuses small for gestational age (SGA), and 13 with fetuses with acute fetal distress in the 3rd trimester, revealing that serum PSG-1 levels were lower in women with complicated pregnancies (Silver *et al*., 1993). We conclude from the study by Sterzik *et al*. (1989) that serum PSG-1 might be an alternative parameter to beta-hCG as a test to predict and evaluate early ectopic gestation. The literature mentioned above indicates that abnormal serum PSG-1 levels may be a determining factor in pregnancy complications.

Our study included 160 volunteers, 80 diagnosed with FGR without any other disease over 24 weeks, and another 80 healthy pregnant women with healthy fetuses. The women with fetuses with FGR had lower serum PSG-1 levels than the healthy pregnant women (48.16±77.46ng/ml and 62.39±103.00ng/ml). However, the difference between the two groups in terms of serum PSG-1 levels was not statistically significant (*p*=0.058).

Examination of the umbilical artery with Doppler ultrasound was performed in 80 patients in the FGR group. The patients were further divided into two groups, one with 29 patients with absent end diastolic flow in umbilical artery Doppler ultrasound at the time of diagnosis and 51 with normal umbilical artery Doppler ultrasound findings. Serum PSG-1 values were lower in the group with absent end diastolic flow (43.86±66.39 *vs*. 50.60±83.63). However, the difference was not statistically significant (*p*=0.964).

Patient demographic characteristics showed the groups were not statistically different in terms of age, gravity, parity, and body mass index. However, the subjects in the FGR group without disorders in Doppler ultrasound examination had a moderately significant correlation between PSG-1, age, and body mass index (*p*<0.05). The two groups were statistically different in terms of weeks of gestation and birth weight (*p*<0.001).

Our study did not find a linear relationship between FGR and PSG-1 levels, but PSG-1 values were lower on average in pregnant women diagnosed with FGR. Likewise, pregnant women with fetuses with FGR and absent end diastolic flow had lower PSG-1 levels than their counterparts with normal Doppler ultrasound findings, although the difference was not statistically significant. This is possibly due to the limited number of participants.

The patients included in the study were in the third trimester or pregnancy. Interestingly, serum PSG-1 levels in the early weeks of pregnancy appear to be more useful in predicting FGR. A scoring system combining other molecules and findings associated with FGR should also be considered. Maybe PSG-1 should be examined in early-onset severe FGR cases in other studies. We believe that assessing PSG-1 levels may be meaningful, especially in patients with fetuses with FGR and disorders in Doppler ultrasound examination.

Prospective studies looking into early trimester pregnancies, including isolated small-for-gestational-age babies, and evaluating newborn postpartum findings should also be considered. In addition to affecting morbidity and mortality, FGR must be followed because of the significant care costs it may produce. For this reason, diagnostic and therapeutic studies are still ongoing. Further studies on FGR are needed since a biochemical marker to diagnose and regulate the condition has not been found.

## CONCLUSION

In conclusion, the measurement of maternal PSG-1 levels may provide clinical information and serve as a predictor of fetal disorders associated mainly with placental problems. Maternal serum PSG-1 levels were lower in women with fetuses with FGR than in healthy pregnant women. Further studies were needed to uncover the role of PSG-1 and other placental proteins.
